# Size Effects in the Verwey Transition of Nanometer-Thick Micrometer-Wide Magnetite Crystals

**DOI:** 10.1021/acs.jpcc.2c03391

**Published:** 2022-08-09

**Authors:** Adolfo del Campo, Sandra Ruiz-Gómez, Eva M. Trapero, Cecilia Granados-Miralles, Adrián Quesada, Michael Foerster, Lucía Aballe, José Emilio Prieto, Juan de la Figuera

**Affiliations:** † Instituto de Cerámica y Vidrio, CSIC, Madrid E-28049, Spain; ‡ Max-Planck-Institut für Chemische Physik fester Stoffe, Dresden 01187, Germany; § Instituto de Química Física “Rocasolano”, CSIC, Madrid E-28006, Spain; ∥ Alba Synchrotron Light Facility, Cerdanyola del Valles E-08290, Spain

## Abstract

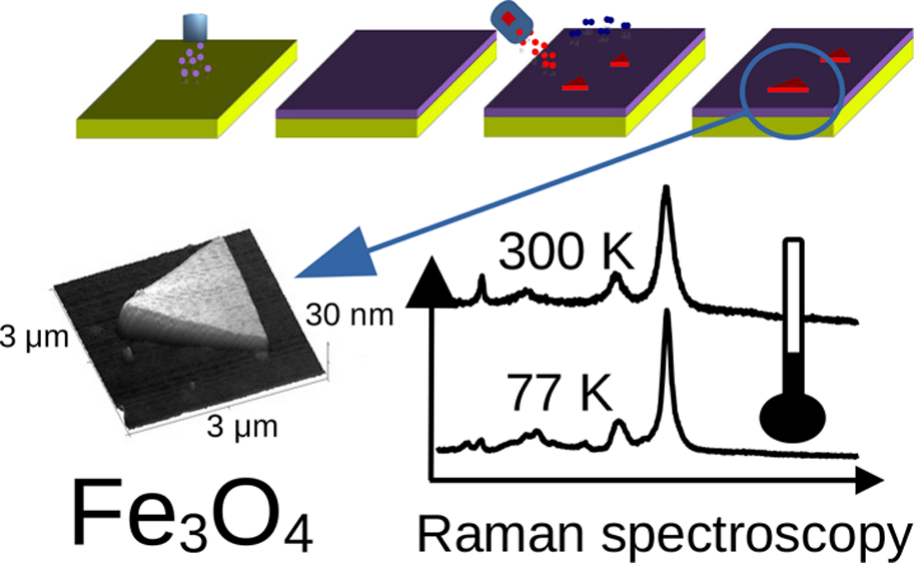

We have monitored the Verwey transition in micrometer-wide, nanometer-thick magnetite islands on epitaxial Ru films on Al_2_O_3_(0001) using Raman spectroscopy. The islands have been grown by high-temperature oxygen-assisted molecular beam epitaxy. Below 100 K and for thicknesses above 20 nm, the Raman spectra correspond to those observed in bulk crystals and high-quality thin films for the sub-Verwey magnetite structure. At room temperature, the width of the cubic phase modes is similar to the best reported for bulk crystals, indicating a similar strength of electron–phonon interaction. The evolution of the Raman spectra upon cooling suggests that for islands thicker than 20 nm, structural changes appear first at temperatures starting at 150 K while the Verwey transition itself takes place at around 115 K. However, islands thinner than 20 nm show very different Raman spectra, indicating that while a transition takes place, the charge order of the ultrathin islands differs markedly from their thicker counterparts.

## Introduction

Magnetite is a mixed-valence iron oxide with a cubic, inverse spinel crystalline structure at room temperature. Magnetite undergoes a phase transition, the Verwey transition,^1^ upon cooling at a temperature of 120 K for bulk crystals. Below the phase transition, the resistivity increases by 2 orders of magnitude, the magnetic anisotropy increases, and the crystallographic structure changes to monoclinic. The origin of these changes has been debated for a century, and this has pushed forward developments in solid-state physics such as the study of the Mott transition. Many details of the transformation, such as the symmetry of the unit cell or the type of charge order at low temperatures, have been the subject of heated debates.^2,3^ Recently, some consensus has been reached on a detailed atomic model of the low-temperature phase based on high-resolution X-ray diffraction data.^4^ From this model, a complex charge order has been suggested, composed of an arrangement of so-called trimeron units: Fe^3+^–Fe^2+^–Fe^3+^ linear chains. However, many details of the phase transition continue to be debated. In particular, given the trend towards the study of materials in nanostructure form in general and for applications in spintronics of magnetite in particular, the effect of the particle size on the Verwey transition is still an open question. Several studies indicate that for nanoparticles smaller than 20 nm the transition is suppressed and disappears completely for sizes smaller than 6 nm.^5^ Size effects such as a decrease of the Verwey temperature have also been detected in films for thicknesses below 60 nm.^6^

The Verwey phase transition can be followed using very different techniques. Most of them are not sensitive to a particular charge order but are rather more indirect. For example, measurements of the resistivity or magnetization changes average the information arising from large ensembles of atoms. Other techniques are of local character, such as Mössbauer spectroscopy or Raman spectroscopy. Mössbauer spectroscopy, due to the low signal-to-noise ratio, is not well suited to study nanostructures. In contrast, Raman spectroscopy has the advantage that it can be applied to small areas of a sample. The usefulness of Raman spectroscopy to follow the Verwey transition was already shown in the classic work of J. L. Verble.^7^ Raman spectroscopy continues to be applied to study the Verwey transition on the surface of bulk crystals.^8−10^ Often sharp changes in some Raman modes are detected as well as an increase of the background intensity,^10^ attributed to the opening of the band gap observed in photoemission spectroscopy.^11^ More recently, Raman spectroscopy has been used to study magnetite films grown on several oxide^12−14^ and metal^15^ crystals.

In the present work, we report on the observation with Raman spectromicroscopy of the Verwey transition of magnetite microcrystals grown on thin epitaxial Ru films. The magnetite crystals possess a high quality as judged from their structural and magnetic properties: they have mostly triangular shapes with typical lateral sizes of several micrometers and heights ranging from a few nanometers up to 100 nm, and they have an excellent order as evidenced by low-energy electron diffraction. Their magnetic properties resemble those of bulk magnetite^16^ and present well-defined magnetic domains already for nanometer thicknesses.^17^

## Methods

The Ru films were deposited on Al_2_O_3_(0001) single-crystal substrates in a home-built magnetron sputtering system. The typical dc magnetron power used was 20 W after 10 min presputtering of a 2” Ru target from Evochem GmbH. The substrates were kept at 900 K during film growth. The films were then transferred to an ultrahigh-vacuum chamber containing a low-energy electron microscope and annealed at temperatures of up to 1300 K. Both the Elmitec III LEEM at the Instituto de Química Física “Rocasolano” and the Elmitec SPELEEM at the CIRCE beamline of the ALBA Synchrotron^18^ were used for the growth of the magnetite islands. The growth was performed by introducing a pressure of 10^–6^ mbar of molecular oxygen, while the substrate was kept at a temperature of 1073 K, and Fe was deposited from a homemade doser consisting of a Fe rod 5 mm in diameter inside a water jacket heated by electron bombardment from a W filament with a typical power of 25 W. X-ray-based characterization using photoemission microscopy was performed at the ALBA Synchrotron CIRCE beamline.

The Raman spectra were acquired with a commercial Renishaw Witec Alpha 300RA confocal Raman spectrometer using a 20× objective with a numerical aperture of 0.4. The light source was a 532 nm laser operated at 1 mW power, selected in order to avoid oxidation of the samples. The spectra presented are the average of 5 scans, each acquired with a 30 s integration time. Measurements were performed at different temperatures between room temperature and liquid nitrogen temperature (77 K). The focus was adjusted at each temperature. We have fitted the Raman spectra by a sum of Lorentzians including a third-degree polynomial for the background after applying a fifth-order median filter.

## Results and Discussion

**Figure 1:**
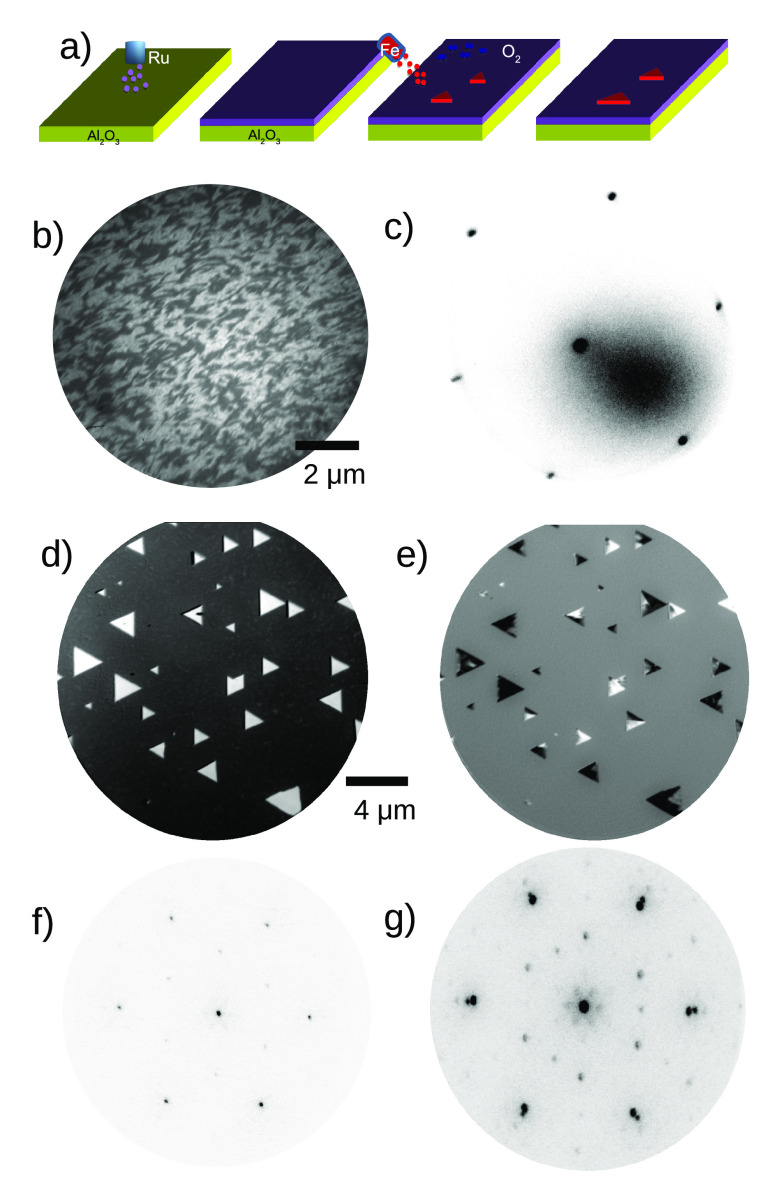
Growth of the magnetite microcrystals. (a) Schematics of the procedure, showing the growth by sputtering of the Ru film and the subsequent growth of the magnetite islands on top by high-temperature oxygen-assisted molecular beam epitaxy. (b) Low-energy electron microscopy image showing the Ru film surface in dark field mode (successive atomic terrace show alternately dark–light gray contrast). (c) Low-energy electron diffraction (LEED) pattern of the Ru film. (d) X-ray absorption image acquired at the white line of the Fe L_3_ absorption edge. (e) X-ray magnetic circular dichroism image of the same region. (f) LEED pattern from the wetting layer. (g) LEED pattern from one of the magnetite islands.

The sample growth process is schematically depicted in Figure 1a; it is similar to the procedure employed by Flege and co-workers to grow ceria islands on Ru films.^19^ Despite having a higher density of steps, see Figure 1b, compared with the Ru single crystals we have used before,^16^ the films are well ordered and single crystalline as previously characterized^20^ and as shown by the diffraction pattern in Figure 1c. The magnetite crystals themselves were grown on top of the Ru films by oxygen-assisted high-temperature molecular beam epitaxy under in situ observation by low-energy electron microscopy. The growth of iron oxide proceeds in the same way as for single-crystal Ru(0001) substrates;^21^ it has been characterized by microspot low-energy electron diffraction as well as X-ray photoemission, X-ray absorption spectroscopy, and X-ray magnetic circular dichroism in photoemission microscopy.^16,17,22^ The crystals grow on top of a wetting layer composed of two atomic layers of FeO^23^ that first covers the Ru substrate and then 3-dimensional islands of magnetite nucleate and grow on top. An X-ray absorption image acquired at the Fe L_3_ edge is shown in Figure 1d. The crystals appear as white triangles as they contain more Fe than the surrounding FeO film. X-ray magnetic circular dichroism images reveal the magnetic domains of the islands (Figure 1e), while the FeO areas in between are not magnetic at room temperature. The diffraction pattern of the islands also shows the distinctive 2 × 2 spots characteristic of the (111) magnetite surface^24^ and their good crystallinity.

**Figure 2:**
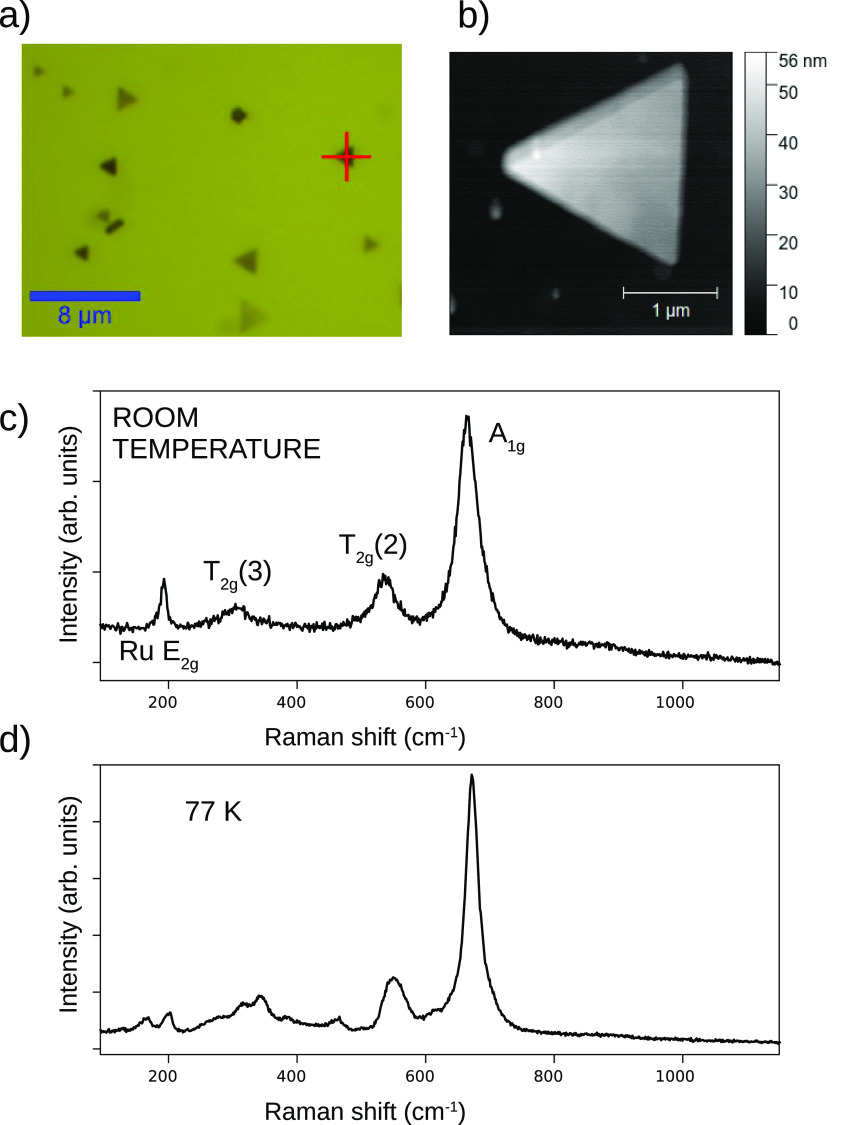
(a) Optical microscopy image of magnetite triangles on a Ru film. (b) Atomic force microscopy image of one of the islands. (c and d) Raman spectra acquired on the island shown in b at room and low temperature, respectively.

The film was then taken out of ultrahigh vacuum and studied ex situ by confocal microscopy and Raman spectroscopy. In Figure 2a, we show an optical micrograph of a region containing a few magnetite crystals. Atomic force microscopy of one of the islands, marked with a red cross, reveals that across its triangular shape, with a 2 μm side, the height varies from 30 to 50 nm. The room-temperature and low-temperature (77 K) Raman spectra acquired on the island are shown in Figure 2c and 2d, respectively.

Magnetite has the cubic inverse spinel structure at room temperature,^25^ which corresponds to the *Fd*3̅*m* space group. All of the Fe^2+^ cations and one-half of the Fe^3+^ cations are located at octahedral sites, while the remaining Fe^3+^ cations occupy tetrahedral sites. The rhombohedral unit cell contains only 14 atoms: 2 tetrahedral iron, 4 octahedral iron, and 8 oxygen atoms. A group theoretical analysis of the structure predicts 42 vibrational modes, of which 5 are Raman active, those with symmetries *A*_1*g*_, *E*_*g*_, and the 3 different *T*_2*g*_.^26^ All of these modes correspond to breathing modes of the tetrahedral cations. The most intense mode is the *A*_1*g*_ one, observed at ω = 665 cm^–1^, which corresponds to the symmetric breathing of the oxygen anions around each tetrahedral cation. The other modes found for magnetite are two of the *T*_2*g*_ ones, at 310 and 535 cm^–1^, respectively. In line with other studies of thin films^13^ we do not observe either of the other two possible modes which are usually very weak, a *T*_2*g*_ mode at 205 cm^–1^ and the *E*_*g*_ one at 380 cm^–1^. The large mode at 192 cm^–1^ can be assigned to the Ru film, as it is detected also in regions were no magnetite crystals are observed. It corresponds to the Ru *E*_2*g*_ transverse optical phonon arising from the shear of the two sublattices of the hcp unit cell.^27^ Such mode has been observed in Ru films and multilayers^27,28^ as well as in other hcp metals.^29^

The spectra of the other islands are very similar at room temperature. The fwhm (Γ) of the modes shown in Figure 2c are, respectively, 38, 41, 118, and 11 cm^–1^ for the magnetite *A*_1*g*_, *T*_2*g*_(2), and *T*_2*g*_(3) and Ru *E*_2*g*_ modes. The spinel modes are much wider than, for example, that of the underlying Ru film. This has been noted in all previously published studies of magnetite. The main contribution to this larger width has been attributed by Verble^7^ to electronic disorder from the random arrangement of Fe^2+^ and Fe^3+^ ions on the B sites. Gupta et al.^9^ explained it in terms of a strong electron–phonon interaction related to the decay of phonons into electron–hole pairs. In such case, the strength of the electron–phonon interaction at room temperature (i.e., in the disordered state with Fe^2+^ and Fe^3+^ ions on the B sites) can be estimated from the width of a given Raman mode according to
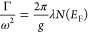
where *g* is the degeneracy of the mode and λ is the intensity of the electron–phonon interaction.^9^ We have measured these values for islands with thickness between 10 and 60 nm and found no clear dependence on the island height. This suggests that the short-range order does not differ appreciably between islands. We present in Table 1 the average values of the relevant parameters together with their statistical errors, determined from six different islands with heights in the 10–60 nm range. In Table 2, we include a comparison of our estimations for the electron–phonon interaction strength with published results.

**Table 1:** Wavenumbers ω (cm^–1^), fwhm Γ (cm^–1^), Γ/ω^2^ (eV), and Strength λ of the Electron–Phonon Interaction Estimated from the Raman Peaks of Each Mode Measured at Room Temperature, Averaged over Several Crystals of Micrometer Width and Height in the Range 10–60 nm

Raman mode	*A*_1*g*_	*T*_2*g*_(2)	*T*_2*g*_(3)
ω (cm^–1^)	664.7 ± 0.7	534 ± 2	311 ± 10
Γ (cm^–1^)	38 ± 1	46 ± 6	81 ± 45
Γ/ω^2^ (eV^–1^)	0.69 ± 0.02	1.3 ± 0.2	7 ± 3
λ	0.037 ± 0.001	0.14 ± 0.02	1.05 ± 0.5

**Table 2:** Values for the Electron–Phonon Interaction Strength for the Main Three Modes Reported in the Literature^a^

	λ_*A*_1*g*__	λ_*T*_2*g*_(2)_	λ_*T*_3*g*_(3)_
this work			
Ru/Al_2_O_3_	0.037 ± 0.001	0.14 ± 0.02	1.05 ± 0.5
bulk single crystal			
ref ^7^	0.034*		
ref ^8^	0.038*		
ref ^9^	0.045	0.20	0.51
thin films			
MgO(100)^12^	0.047	0.33	0.98
MgO(100)^13^	0.035*	0.11*	1.06*
Al_2_O_3_(0001)^13^	0.035*	0.13*	1.01*
TiN/Si(100)^30^	0.039*	0.456	

a Values marked with an asterisk have been estimated from the data provided in the corresponding reference.

The electron–phonon interaction is much weaker for the *A*_1*g*_ mode than that for the *T*_2*g*_(2) one, which in turn is an order of magnitude smaller than that for the *T*_2*g*_(3) mode. We note that our results are comparable to those reported for the best films,^13^ where it is mentioned that the samples were selected for their high Verwey temperature. That the interaction with the *T*_2*g*_ modes is much higher has been suggested to be due to sharing the symmetry of electronic states at the Fermi level.^9^ However, there is no clear explanation for the reason it should be so different between the two *T*_2*g*_ modes. Phase el al.^12^ attributed it to the presence of antiphase boundaries. While we expect that our crystals, having grown each presumably from a single nucleus, lack antiphase boundaries, we note that the films reported by Yazdi^13^ should contain a large number of antiphase boundaries, but they present (together with our results) the lowest values for thin films.

The spectrum at low temperature (Figure 2d) shows many more peaks. This is a distinctive feature of the Verwey transition in magnetite.^7^ The spectra of the islands are very similar to those reported below the Verwey transition both for magnetite single crystals^7,8,10^ and for high-quality films.^12,13^ We take this as proof of the occurrence of the Verwey transition in our micrometer-wide, nanometer-thick magnetite islands.

**Figure 3:**
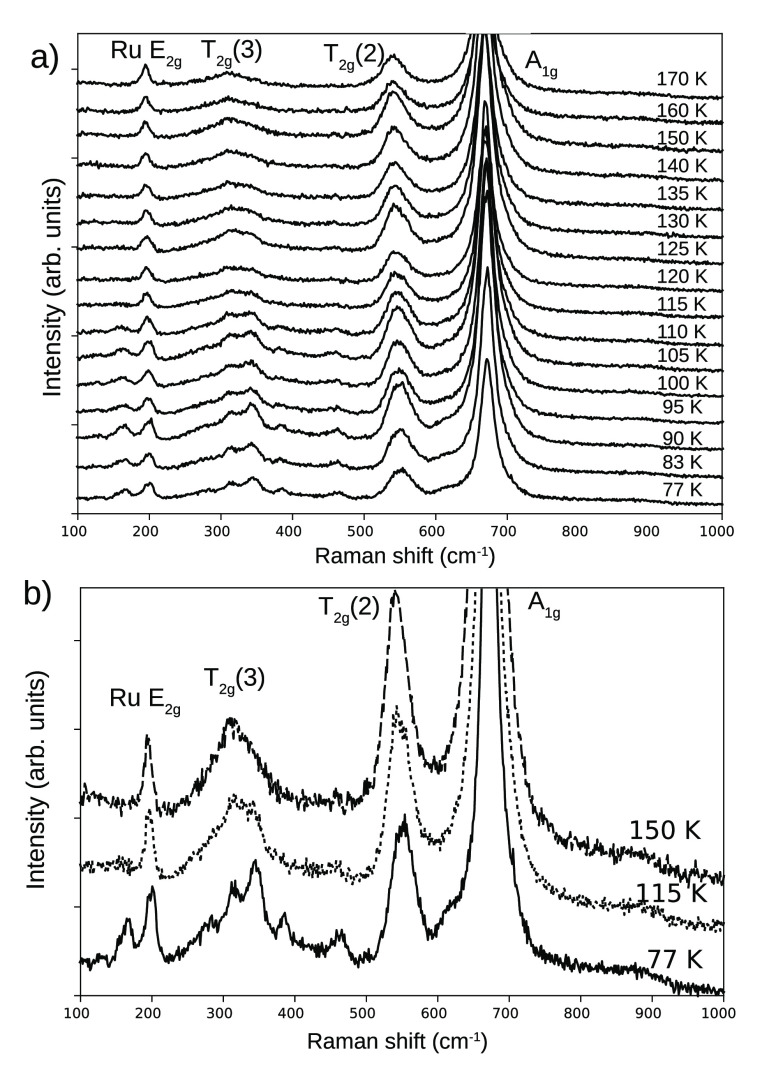
(a) Raman spectra acquired through the Verwey transition in the range 170–77 K. (b) Raman spectra at three representative temperatures to show the characteristics of the evolution with temperature (150, 115, and 77 K), acquired on the island displayed in the previous figure.

The detailed temperature evolution of the Raman spectrum of the island shown in Figure 2 is presented in Figure 3. The evolution from the room-temperature to the sub-Verwey spectra occurs in several stages. In the first one, in the range between 170 and 115 K, mostly the shape of the high-temperature peaks is affected. For example, a shoulder appears on the right-hand side of the *T*_2*g*_(3) mode. Upon further cooling, new peaks appear at 160 and 480 cm^–1^, the *T*_2*g*_(3) mode at 310 cm^–1^ breaks into several peaks, and a shoulder appears at the left side of the *A*_1*g*_ mode.

There are no reported first-principle studies of the modes of magnetite using the atomic positions of the trimeron model.^4^ However, some of the changes can be understood qualitatively. For example, while there is only a single tetrahedral site in the high-temperature cubic phase, in the monoclinic structure^4^ there are eight different tetrahedral sites. A study^8^ of the expected modes has been done with a simpler orthorhombic unit cell with *Pmca* symmetry in which there is a doubling of the unit cell along one axis while the other axis contains the diagonals of the cubic cell. In this case, 78 Raman active modes are expected, instead of the 5 found for the cubic phase. Thus, obviously many more peaks should and in fact do appear.

Some modes should persist with little changes across the Verwey transition. The cubic *A*_1*g*_ mode is a prime example. In the low-temperature phase, it corresponds to an *A*_*g*_ mode, with which it shares the same Raman tensor. Other modes arise from the structural change from the cubic to the monoclinic unit cell. The *T*_2*g*_ modes should split into *B*_1*g*_ + *B*_2*g*_ + *B*_3*g*_ modes in the monoclinic phase. The modes arising from the high-temperature cubic ones have been named “shoulder” modes.^13^ Such is the peak at 615 cm^–1^. These modes are then appropriate to follow the structural changes in the unit cell of magnetite. In addition, there are modes which are expected to arise due to the onset of the charge ordering below the Verwey transition, as has been observed in other oxides.^31^ Those modes are unrelated to any one of the cubic phase. Their origin is attributed to a substantial electrical polarization arising from off-center atomic displacements in the trimeron charge-ordered phase. The modes at 160 and 480 cm^–1^ belong to this group. They do not reflect the structural modifications but the electronic changes that correspond to the appearance of the charge-ordered state.

Thus, we separate the Raman peaks into three groups: those that do not show a large change across the transition, those that split into new peaks (which reflect the change in the unit cell), and the new peaks that arise from the appearance of the charge-ordered state. We discuss the evolution of peaks belonging to each group separately in order to take advantage of the power of Raman spectroscopy to track the different changes occurring in magnetite through the Verwey transition. In thin films, structural changes have been observed to precede the charge-order appearance when lowering the temperature.^13^

**Figure 4:**
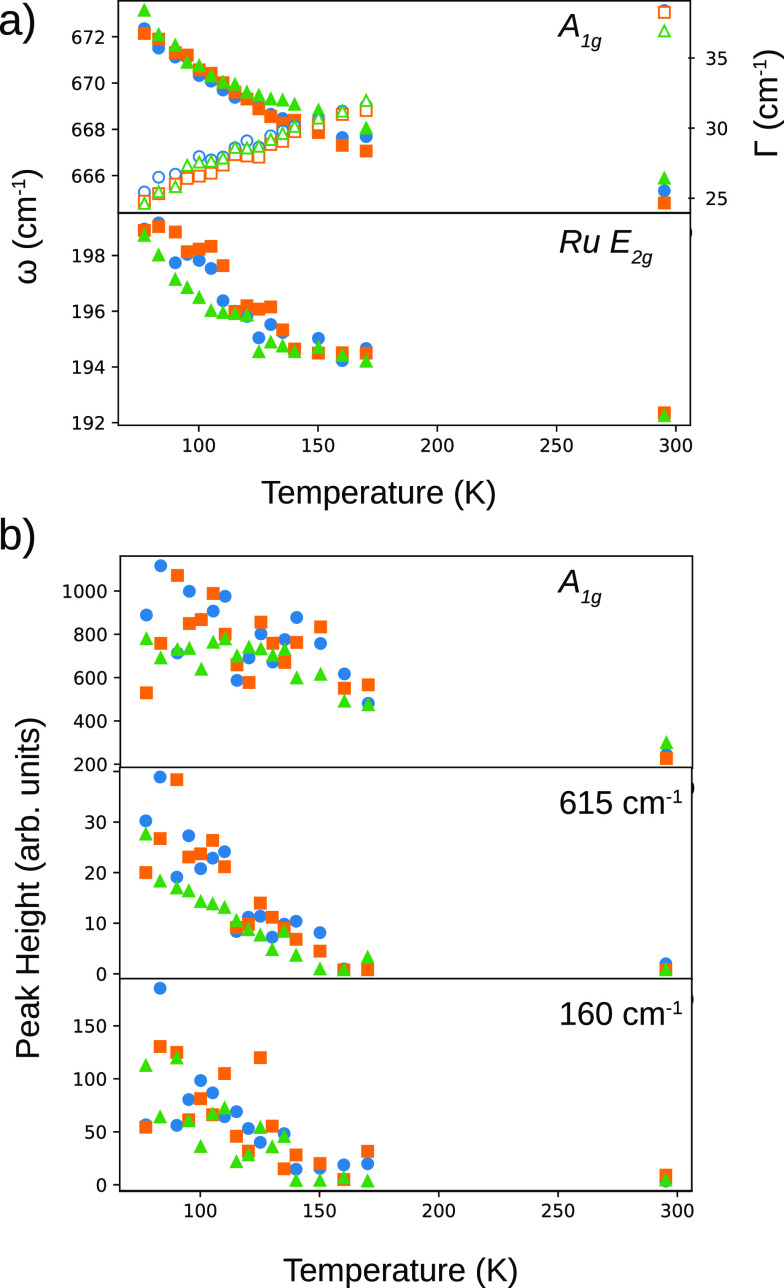
Evolution with temperature of (a) (top) ω and Γ of the *A*_1*g*_ mode and (bottom) ω of the Ru *E*_2*g*_ mode. (b) Peak height of the *A*_1*g*_ Raman mode (top), mode at 615 cm^–1^ (middle), and mode at 160 cm^–1^ (bottom). Blue circles, orange squares, and green triangles correspond to different islands which are 38 ± 5, 37 ± 14, and 62 ± 20 nm thick, respectively.

The most intense mode is the cubic *A*_1*g*_ mode, which belongs to the first group. As Verble^7^ already indicated, it does not drastically vary across the Verwey transition but smoothly changes its position and width (see Figure 4a). The evolution is similar to that reported for a high-quality thin film.^13^ Other works report sharp changes in its width and position,^8,12^ which we do not observe. It is worth noting that the evolution of *A*_1*g*_ is very similar to that of the Ru film mode. In general, Raman modes are expected to shift to lower frequencies upon heating. This is due to anharmonicity, also causing thermal expansion and changes in the population of the vibrational energy levels with increasing temperature. That the magnetite *A*_1*g*_ mode, which reflects the symmetric motion of the oxygen atoms around the Fe tetrahedral cations, follows the same evolution in our islands suggests that the thermal expansion is not severely affected by the Verwey transition.

The *T*_2*g*_ modes behave differently: their shape clearly changes in the range between 150 and 115 K, reflecting the onset of a structural transition. This is also nicely shown by the shoulder mode at 615 cm^–1^.^13^ As seen in Figure 4b, the increase in intensity at this energy starts already at 150 K.

The modes in the third group, such as the one at 160 cm^–1^, which signal the onset of the charge-ordered state, do not appear until the temperature is further lowered (see Figure 4b, bottom). We interpret this as meaning that the Verwey temperature of our crystals corresponds to the appearance of these latter peaks, following Yazdi et al.^13^ As in their case, this implies that the structural changes precede the electronic ones upon lowering the temperature.

**Figure 5:**
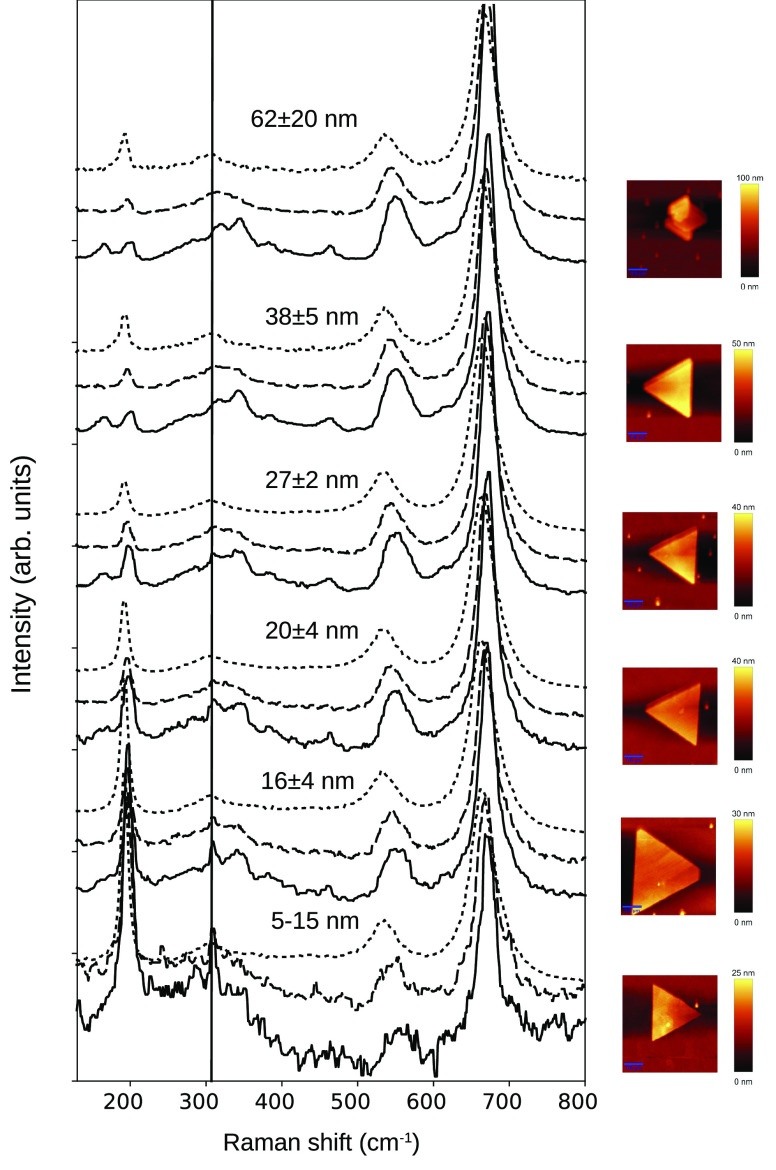
Raman spectra of islands of different thickness at room temperature (dotted line), at 115 K (dashed line), and at 77 K (continuous line). Atomic force microscopy images of each island are shown at the right-hand side. The line marks the location of the sharp feature observed on the thinner islands at low temperature.

Finally, we discuss the full low-temperature spectrum as a function of island height, as shown in Figure 5. The spectra of islands with thicknesses above 20 nm show the same features. In all cases, there is a smooth evolution down to 115 K; below this, we find the sudden appearance of the modes attributed to the sub-Verwey charge order. For crystals thinner than 20 nm the behavior changes, and for the thinnest crystal (between 5 and 15 nm, depending on the crystal side) the low-temperature spectrum, while clearly different from the room-temperature one, also differs completely from the reference spectra of sub-Verwey magnetite. There is a very sharp new mode that only appears at low temperatures at 300 cm^–1^ (marked with a blue line in the figure), which is already observed for a thickness of 20 nm. We note that the location of that mode corresponds to the room-temperature *T*_2*g*_(3) one. While the room-temperature mode is wide for all islands (as discussed above), the low-temperature feature is rather sharp. As that peak arises from an asymmetric breathing mode which corresponds in one direction to the motion of the oxygen cations within the (111) film plane while in the other to motion in a direction making an angle to that plane, it is tempting to relate this observation to some confinement effect of the *T*_2*g*_(3) mode, as has been reported for Co/Ru films.^27^ However, we note that we have not observed at room temperature any confinement effect whatsoever, i.e., we observed no shift in the mode as a function of size. One possibility is that small shifts might be masked by the large width arising from the high electron–phonon interaction strength. Another surprising detail is that the position of this feature corresponds to the room-temperature mode but not to the expected one at low temperature, as observed in thicker films. We note that the origin of the observed feature might well be related to a near-surface Verwey phase transition and thus would reflect the larger contribution of the near-surface region in ultrathin magnetite islands. Further work will be needed to fully characterize this regime.

In any case, we thus have shown that the charge order of the crystals is similar to that of bulk magnetite only for thicknesses larger than 20 nm. For thinner crystals, the Raman spectra differ drastically from the high-temperature cubic ones, indicating that there is a phase transition in which the properties of the magnetite crystal change, even if the transition to the bulk-like trimeron charge-ordered state does not take place. Regrettably, we lack at present a detailed microscopic characterization of the crystallographic structure of such islands. Work to determine such structure is planned.

## Conclusions

We have measured Raman spectra of magnetite microcrystals with thicknesses of tens of nanometers. At room temperature, they correspond to the typical spectra of bulk magnetite. Our estimates of the electron–phonon interaction strength are similar to the values for the best single crystals and thin films reported in the literature. At 77 K, the spectra of crystals thicker than 20 nm correspond to those reported for the low-temperature phase of bulk magnetite. With decreasing temperature, down to 115 K, first, the high-temperature phase peaks corresponding to the *T*_2*g*_ modes change in shape. We do not observe any abrupt change in the *A*_1*g*_ mode. Below 115 K, peaks corresponding to the charge order in the low-temperature phase of magnetite appear. However, for crystals thinner than about 20 nm, our results suggest that although a transition to a new phase takes place, its charge order and structure differ from those characteristic of the bulk material, highlighting the role of size effects in the Verwey transition.
